# The Role of T Cell Immunoglobulin Mucin Domains 1 and 4 in a Herpes Simplex Virus-Induced Behçet's Disease Mouse Model

**DOI:** 10.1155/2013/903948

**Published:** 2013-12-26

**Authors:** Ju A. Shim, Eun-So Lee, Bunsoon Choi, Seonghyang Sohn

**Affiliations:** ^1^Laboratory of Cell Biology, Ajou University Institute for Medical Sciences, Suwon 443-721, Republic of Korea; ^2^Department of Dermatology, Ajou University, School of Medicine, Suwon 443-721, Republic of Korea; ^3^Brain Korea 21 Project for Medical Science, Suwon 443-721, Republic of Korea

## Abstract

The T cell immunoglobulin mucin (TIM) proteins regulate T cell activation and tolerance. TIM-1 plays an important role in the regulation of immune responses and the development of autoimmune diseases. TIM-4 is a natural ligand of TIM-1, and the interaction of TIM-1 and TIM-4 is involved in the regulation of T helper (Th) cell responses and modulation of the Th1/Th2 cytokine balance. Behçet's disease (BD) is a chronic, multisystemic inflammatory disorder with arthritic, intestinal, mucocutaneous, ocular, vascular, and central nervous system involvement. Tim-1 expression was lower in a herpes simplex virus-induced BD mouse model compared to that in asymptomatic BD normal (BDN) mice. Tim-4 expression was higher in BD mice than that in BDN mice. In this study, we investigated the Tim expression in a BD mouse model with BD-like symptoms. Tim-1 and Tim-4 expression was regulated by an expression vector or siRNA injected into the BD mouse model. The *Tim-1* vector injected into BD mice resulted in changes in BD-like symptoms and decreased the severity score. Treatment with Tim-4 siRNA also improved BD-like symptoms and decreased the severity score accompanied by upregulation of regulatory T cells. We showed that regulating Tim-1 or Tim-4 affected BD-like symptoms in mice.

## 1. Introduction

The T cell immunoglobulin and mucin domain (TIM) family is located on chromosome 11B1.1 in mice and consists of several members (*Tim*-1–8). In humans it is located on chromosome 5q33.2 and consists of three members (*TIM*-1, 3, and 4) [1]. Individual TIM family members may serve as susceptibility markers for asthma, allergies, and autoimmune diseases, as well as potential cell surface markers for T helper (Th) type 1 and Th2 cells [1, 2]. Therefore, the human *TIM* gene family is critical in the regulation of Th1/Th2 mediated immunological reactions [2].

TIM-1 was first identified as a hepatitis A virus cellular receptor 1 [3, 4] and a kidney injury molecule, KIM-1 [5, 6]. TIM-1 is expressed on CD4^+^ T cells after activation and its expression is sustained preferentially in Th2 but not Th1 cells [1, 7]. TIM-1 plays an important role regulating immune responses and the development of autoimmune disease. The high-avidity anti-Tim-1 antibody enhances the severity of experimental autoimmune encephalitis by increasing autopathogenic Th1 and Th17 responses, whereas the low-avidity antibody inhibits autopathogenic Th1 and Th17 responses [8].

TIM-4 is a natural ligand of TIM-1 [7] and is exclusively expressed on antigen-presenting cells, including dendritic cells (DCs) and macrophages [9, 10], where it mediates phagocytosis of apoptotic cells and plays an important role maintaining tolerance [11, 12]. TIM-1 and TIM-4 interact to regulate Th cell responses and modulate the Th1/Th2 cytokine balance [7]. DC-derived TIM-4 maintains TIM-1 in Th2 cells in a stable status and plays a critical role sustaining Th2 polarization [13]. TIM-4 binding to TIM-1 has different effects on T cell proliferation. A higher dose of Tim-4-Ig consistently leads to an increase in T cell proliferation upon ligation with the T-cell receptor, whereas a lower concentration of Tim-4-Ig inhibits T cell proliferation [7]. Human TIM-1 is also associated with other types of immune dysfunction, such as atopic dermatitis, allergy, rheumatoid arthritis, asthma, and systemic lupus erythematosus (SLE) [14–18], suggesting that Tim-1 may regulate immune responses. In addition, TIM-4 expression in peripheral blood mononuclear cells (PBMCs) also increases in patients with SLE [13].

Behçet's Disease (BD) is a Th1-polarized [19], chronic, multisystemic inflammatory disorder with arthritis, gastrointestinal, mucocutaneous, ocular, vascular, and central nervous system involvement. This disease takes a chronic course with periodic exacerbations and progressive deterioration [20]. The etiology of BD is unclear; however, viral infection has long been postulated as one of the main factors. Since Behçet first proposed a viral etiology [21], his hypothesis has been verified by detecting virus in saliva [22], intestinal ulcers [23], and genital ulcers [24, 25] of patients with BD. Subsequently, herpes simplex virus (HSV) inoculation of the earlobes of ICR mice resulted in the development of BD-like symptoms [26]. Manifestations in mice inoculated with HSV include multiple symptoms such as oral ulcers, genital ulcers, skin ulcers, eye symptoms, intestinal ulcers, arthritis, and neural involvement, as well as skin crusting. The frequencies of these symptoms are similar to those of patients with BD [27].

TIM-1 and TIM-4 have not been studied much in BD until now. In this study, we investigated the Tim expression in a BD mouse model with BD-like symptoms. The expression Tim-1 and Tim-4 was analyzed in BD mice and the changes in BD-like symptoms were observed by regulating of Tim-1 or Tim-4 expression. Furthermore, the changes in cellular phenotypes and cytokine levels on immune cells were confirmed after upregulation of Tim-1 or downregulation of Tim-4 in BD mice.

## 2. Materials and Methods

### 2.1. Antibodies and Reagents

Mouse anti-CD4, anti-Tim-1, anti-Tim-4, anti-CD8a, anti-CD122, anti-CD11b, anti-CD11c, and anti-CD25 antibodies as well as an anti-Foxp3 staining kit were purchased from eBioscience (San Diego, CA, USA).

### 2.2. Animal Experiments

ICR male mice (4-5 weeks old) were infected with HSV type 1 (1 × 10^6^ pfu/mL, F strain) grown in Vero cells as described previously [26]. We used anesthetic composed of a mixture of Zoletil (Virbac Lab, Carros, France) and Rompun (Bayer, Seoul, Korea). The ratio of Zoletil and Rompun was 1 : 4, and it was administered at a dose of 40 *μ*L/mouse (tiletamine 10 mg/kg, zolazepam 10 mg/kg, and xylazine hydrochloride 36 mg/kg) via intramuscular injection. Virus inoculation was conducted twice at 10 day intervals, after which the animals were observed for 16 weeks. Animals were handled in accordance with a protocol approved by the animal care committee of Ajou University School of Medicine (Institutional approved number: AMC-102).

### 2.3. BD-Like Symptoms

Multiple symptoms were observed in the mice after HSV inoculation, and 12% of the HSV-injected mice developed BD-like symptoms. Disappearance of symptoms or a >20% decrease in dimension of lesion size was classified as effective. Determination of the BD severity score was followed by determining the value of the BD activity index, as outlined on the BD activity form (http://www.behcet.ws/pdf/BehcetsDiseaseActivityForm.pdf). Symptoms exhibited by patients, including mouth ulceration, genital ulceration, erythema, skin pustules, skin ulceration, joints-arthritis, diarrhea, blurred or red eye (right, left), reduced vision (right, left), loss of balance, discoloration of skin, and swelling of the face were selected and analyzed in the BD mouse model. The score of each symptom was one, and the total score was used to determine the BD severity score.

### 2.4. *Tim-1* DNA Constructs

A *Tim-1* construct with an extracellular Flag epitope tag was generated [28]. A cDNA clone containing the entire coding sequence of murine *Tim-1* was constructed. Briefly, the *Tim-1 *open reading frame (excluding the start codon and signal sequence) was amplified from this clone by polymerase chain reaction and ligated into a pCDEF3 expression plasmid [29]. All DNA constructs were verified by automated DNA sequencing. All plasmids used were purified by two passes through Endo-Free columns (Qiagen, Chatsworth, CA, USA) as described previously [30].

### 2.5. Preparation of Tim-4 Small Interfering RNA (siRNA)

Tim-4 siRNA (siTim-4) was synthesized by Genolution Pharmaceuticals, Inc. (Seoul, Korea). The synthesized sequences of Tim-4 siRNA were sense: 5′- CUA AAU CAC AUC AGA UCA ACA GCA GUU -3′, and antisense: 5′- CUG CUG UUG AUC UGA UGU GAU UUA GUU -3′. The Tim-4 siRNA with transfection reagent jetPEI (PolyPlus-transfection, Llkirch, France) was used to inject into mice.

### 2.6. *Tim-1* Vector and Tim-4 siRNA Administration of BD Mice

Ten *μ*g of *Tim-1* vector was intraperitoneally injected four times at 2 day intervals into BD mice when the BD-like symptoms appeared, followed by 2 weeks of observations. The control was injected with the pCDEF3 empty vector. Five *μ*g of siRNA Tim-4 was intraperitoneally injected three times at 2 day intervals into BD mice to downregulate Tim-4, followed by a 2-week observation. Scrambled siRNA was used as the negative control (Genolution Pharmaceuticals, Inc., Seoul, Korea).

### 2.7. Flow Cytometry

PBMCs and lymph node cells were isolated from mice and erythrocytes were removed from cell suspensions in ACK solution, then washed with phosphate buffered saline (PBS). The cells were surface-stained with anti-mouse antibodies (CD4, CD8, CD11b, CD11c, CD25, CD122, Tim-1, and Tim-4) for 30 min at 4°C in the dark. An anti-mouse Foxp3-staining buffer set was used according to the manufacturer's instructions to detect Foxp3 intracellularly. Briefly, cells were fixed using Fix/perm buffer after washing with 1× permeabilization buffer and then incubated with anti-mouse Foxp3 antibody. For analysis, the cells were gated and then the population of stained cells was analyzed by a flow cytometer (FACS Canto II; Becton Dickinson, Franklin Lakes, NJ, USA) with ≥10,000 gated lymphocytes.

### 2.8. Enzyme Linked Immunosorbent Assay (ELISA)

Serum was collected 14 days after the first administration of the *Tim-1* vector and Tim-4 siRNA into BD mice. Serum was analyzed using commercial ELISA kits for the detecting mouse interleukin (IL)-6 (R&D Systems, Minneapolis, MN), tumor necrosis factor (TNF)-*α* (R&D Systems), IL-17 (R&D Systems), IL-4 (R&D System), and interferon (IFN)-*γ* (R&D Systems), according to the manufacturer's instructions. The ELISA reader was Bio-Rad model 170–6850 microplate reader, and samples were read at a wavelength of 450 nm.

### 2.9. Statistical Analysis

All data are expressed as mean ± standard deviation. Statistical differences between the experimental groups were determined using Student's *t-*test with a Bonferroni correction. Statistical analysis was conducted using MedCalc version 9.3.0.0. A *P* < 0.05 was considered significant.

## 3. Results

### 3.1. The Frequencies of Tim-1 and Tim-4 Expressing Cells in Normal Healthy, BD Normal (BDN), and BD Mice

**Figure 1:**
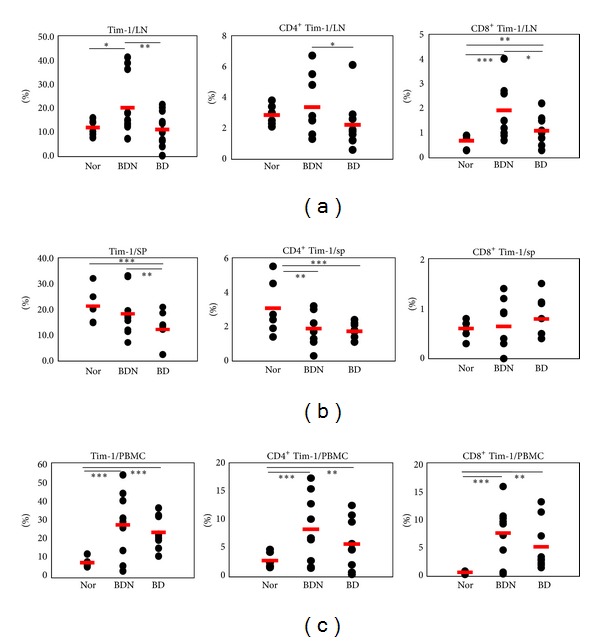
The frequencies of Tim-1^+^ cell phenotypes in normal healthy, Behçet's disease (BD) normal (BDN), and BD mice. The frequencies of Tim-1^+^, CD4^+^Tim-1^+^ and CD8^+^Tim-1^+^, cells in BD mice were compared to Nor and BDN mice by FACS analysis in (a) lymph nodes, (b) spleen, and (c) peripheral blood mononuclear cells (PBMCs) (*n* = 6–12) (*P < 0.1, **P < 0.05, ***P < 0.01). Nor: normal healthy mice, BDN: BD normal mice.

The frequencies of Tim-1^+^, CD4^+^Tim-1^+^, and CD8^+^Tim-1^+^ cells were analyzed in cells from lymph nodes (LN), spleen (SP), and PBMCs of normal healthy (Nor) and BDN (HSV-1 was inoculated but no symptoms) mice and compared with those in BD mice by FACS. In LN, the frequencies of Tim-1^+^, CD4^+^Tim-1^+^, and CD8^+^Tim-1^+^ cells in BD mice were significantly lower than those in BDN mice (BDN versus BD (%): Tim-1^+^, 20.1 ± 11.5 (*n* = 12) versus 11.1 ± 6.7 (*n* = 12), *P* = 0.01; CD4^+^Tim-1^+^, 3.4 ± 1.9 (*n* = 9) versus 2.2 ± 1.6 (*n* = 9), *P* = 0.09; CD8^+^Tim-1^+^, 1.9 ± 1.1 (*n* = 9) versus 1.1 ± 0.6 (*n* = 9), *P* = 0.04) (Figure 1(a)). In SP, the BD mice showed significantly lower frequencies than Nor or BDN mice (Nor versus BDN versus BD (%): Tim-1^+^, 21.3 ± 6.5 (*n* = 6) versus 18.3 ± 8.5 (*n* = 10) versus 12.2 ± 5.9 (*n* = 10), Nor versus BD *P* = 0.006, BDN versus BD *P* = 0.04; CD4^+^Tim-1^+^, 3.1 ± 1.6 (*n* = 6) versus 1.9 ± 1.0 (*n* = 8) versus 1.7 ± 0.5 (*n* = 8), Nor versus BD *P* = 0.02, BDN versus BD *P* = 0.35). CD8^+^Tim-1^+^ cells showed similar frequencies among three groups (Figure 1(b)). In PBMCs, the frequencies of BD mice were slightly lower than those of BDN, but not significantly (BDN (*n* = 9) versus BD (*n* = 9) (%): Tim-1^+^, 27.6 ± 17.1 versus 23.6 ± 8.4,  *P* = 0.54; CD4^+^Tim-1^+^, 8.3 ± 5.9 versus 5.7 ± 4.4, *P* = 0.31; CD8^+^Tim-1^+^, 7.6 ± 5.0 versus 5.2 ± 4.3, *P* = 0.29). BD mice had significantly higher levels than those of Nor mice (Figure 1(c)). These data indicate that the frequencies of Tim-1 expressing cells in BD mice were downregulated compared to those in BDN mice.

**Figure 2:**
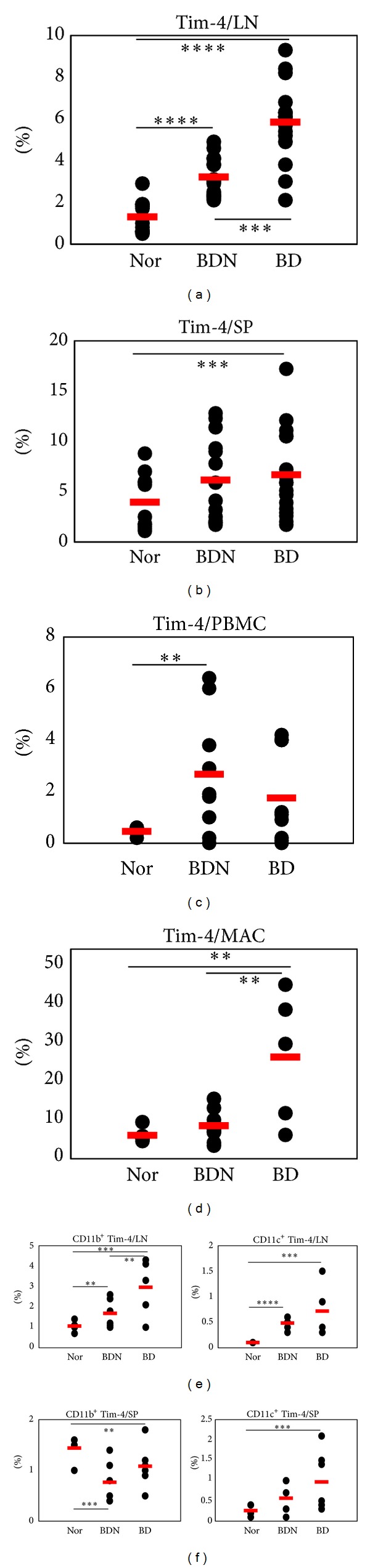
The frequencies of Tim-4^+^ cell phenotypes in normal healthy, BDN and Behçet's disease (BD) mice. The frequencies of Tim-4^+^ cells in BD mice were compared to Nor and BDN mice by FACS analysis in (a) lymph nodes, (b) spleen, (c) PBMCs, and (d) peritoneal macrophages. The expression of CD11b^+^Tim-4^+^ and CD11c^+^Tim-4^+^ cells in BD mice were also compared to those in Nor and BDN mice in (e) lymph nodes and (f) spleen (*n* = 5–17) (**P < 0.05, ***P < 0.01, ****P < 0.001) Nor: normal healthy mice, BDN: BD normal mice.

The frequencies of Tim-4^+^ cells were also analyzed in LN, SP, and PBMCs of Nor, BDN, and BD mice. In LN, the frequencies of Tim-4^+^ cells in BD mice were significantly higher than those in Nor and BDN mice (Nor versus BDN, versus BD (%): 1.3 ± 0.8 (*n* = 11) versus 3.2 ± 0.9 (*n* = 15) versus 5.8 ± 2.0 (*n* = 15), Nor versus BDN *P* = 0.000005, Nor versus BD, *P* = 0.0000001, BDN versus BD *P* = 0.00003) (Figure 2(a)). In SP, the frequencies of Tim-4^+^ cells in BD mice were also significantly higher than those in Nor mice (Nor versus BD (%): 3.9 ± 2.8 (*n* = 11) versus 6.7 ± 4.3 (*n* = 17), *P* = 0.035). However, the difference between BDN and BD mice was not significant (Figure 2(b)). In PBMCs, the frequencies of Tim-4^+^ cells in BD mice were higher than those in Nor mice (Figure 2(c)). Moreover, in peritoneal macrophages, the frequencies of Tim-4^+^ cells in BD mice were significantly higher than those in Nor and BDN mice (Nor versus BDN versus BD (%): 6.1 ± 2.0 (*n* = 5) versus 8.6 ± 4.0 (*n* = 9) versus 26.6 ± 17.0 (*n* = 5), Nor versus BDN,  *P* = 0.11, Nor versus BD,  *P* = 0.02, BDN versus BD,  *P* = 0.005). No differences between Nor and BDN mice were observed (Figure 2(d)). In addition, we also examined the frequencies of CD11b^+^Tim-4^+^ and CD11c^+^Tim-4^+^ cells in LN and SP. In LN, both markers in BD mice were higher than those in Nor and BDN mice (Nor (*n* = 5) versus BDN (*n* = 6) versus BD (*n* = 5) (%): CD11b^+^Tim-4^+^, 1.1 ± 0.3 versus 1.7 ± 0.7 versus 3.0 ± 1.4, Nor versus BDN,  *P* = 0.05, Nor versus BD,  *P* = 0.01, BDN versus BD,  *P* = 0.04; CD11c^+^Tim-4^+^, 0.1 ± 0.0 versus 0.5 ± 0.1 versus 0.7 ± 0.5, nor versus BDN,  *P* = 0.00003, Nor versus BD,  *P* = 0.01, BDN versus BD,  *P* = 0.14) (Figure 2(e)). In splenocytes, expression of CD11c^+^Tim-4^+^ cells was similar to that in LN in Nor (0.3 ± 0.1%, *n* = 5), BDN (0.6 ± 0.4%, *n* = 6)  and BD mice (1.0 ± 0.6%, *n* = 8) (Nor versus BDN,  *P* = 0.07, Nor versus BD,  *P* = 0.07, BDN versus BD,  *P* = 0.1). In contrast, the frequencies of CD11b^+^Tim-4^+^ cells in BD mice were significantly lower than those in Nor, but higher than those in BDN mice (Figure 2(f)). These data suggest that the frequencies of Tim-4 expressing cells in BD mice were upregulated compared to those in BDN mice.

### 3.2. Administration of the *Tim-1* Vector Upregulates the Frequencies of Tim-1^+^ Cells In In Vivo LN

**Figure 3:**
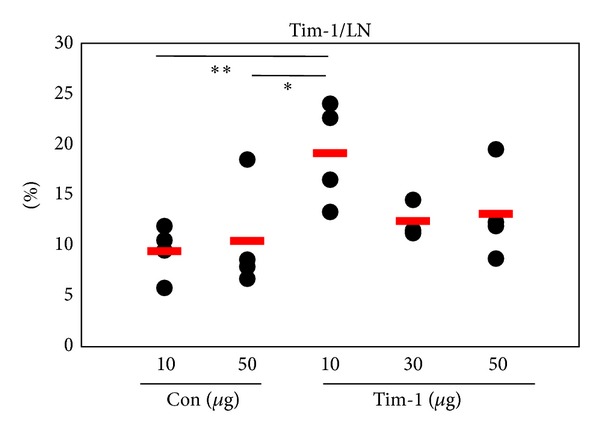
Administration of *Tim-1* vector increased the frequencies of Tim-1^+^ cells. The *Tim-1* vector was injected intraperitoneally at 10, 30, and 50 *μ*g/mouse into Nor mice twice at 2 day intervals to observe up-regulation of Tim-1 expression. The day of the last injection, the frequencies of Tim-1^+^ cells in LN were compared to the control vector injected group by FACS analysis. The control was injected with the pCDEF3 empty vector (10 or 50 *μ*g/mouse) (*n* = 3-4) (**P < 0.05). Con: control vector injection to normal (Nor) mice, Tim-1: *Tim-1* vector injection to Nor mice.

We investigated the change in BD-like symptoms according to the regulation of Tim-1 expression. The *Tim-1* expression vector was administered to BD mice to upregulate Tim-1 expression. The *Tim-1* vector was injected intraperitoneally at 10, 30, and 50 *μ*g/mouse into Nor mice twice at 2 day intervals. The frequencies of Tim-1^+^ cells from LN were compared to those of the control vector injected group the day after the last injection. The expression of Tim-1^+^ cells in the 10 *μ*g *Tim-1* vector injected group (19.1 ± 5.1%, *n* = 4) was significantly higher than that in the control vector injected group (10 *μ*g: 9.4 ± 2.6%, *n* = 4, *P* = 0.01; 50 *μ*g: 10.4 ± 5.4%, *n* = 4, *P* = 0.06) (Figure 3). However, the 30 (*n* = 3) and 50 *μ*g (*n* = 4) *Tim-1* vector injected groups showed lower expression than that in the 10 *μ*g injected group. Thus, 10 *μ*g of the *Tim-1* vector was used for subsequent experiments.

### 3.3. Administration of *Tim-1* Vector Affected BD-Like Symptoms

**Figure 4:**
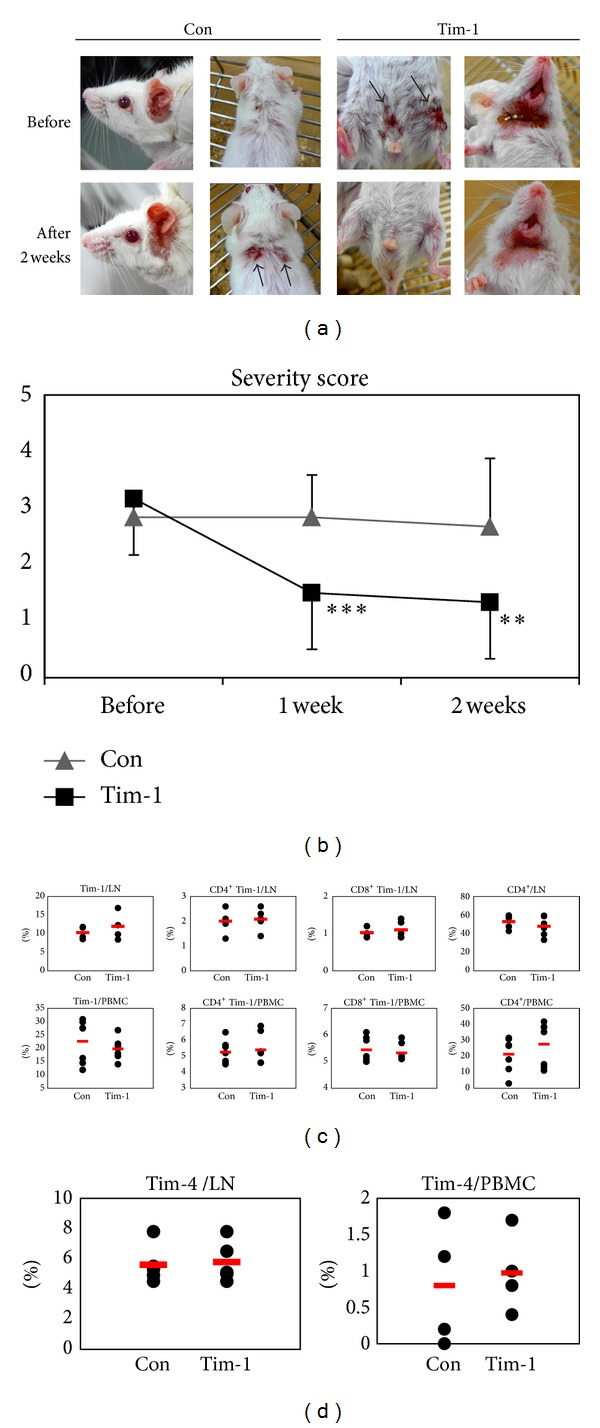
Administration of Tim-1 improved the Behçet's disease (BD)-like symptoms, but the frequencies of Tim-1 expressing cells remained unchanged. Ten *μ*g/mouse of *Tim-1* vector was injected intraperitoneally four times at 2 day intervals into BD mice, followed by 2 weeks of observations. (a) Photographs of mice were taken before and at 1 and 2 weeks after the first treatment of the *Tim-1* vector and control vector injected groups. (b) Severity scores were compared before and at 1 and 2 weeks after treatment. (c) Two weeks after first *Tim-1* vector injection, the frequencies of Tim-1^+^, CD4^+^Tim-1^+^, CD8^+^Tim-1^+^, CD4^+^ T cells, and (d) Tim-4^+^ cells in LN and PBMCs were evaluated by FACS analysis (*n* = 5-6) (*P < 0.1). Con: control vector injection to BD mice, Tim-1: *Tim-1* vector injection to BD mice.

Ten *μ*g of *Tim-1* vector was injected intraperitoneally into BD mice four times at 2 day intervals, followed by observations for 2 weeks. Skin and genital ulcers improved after administering the *Tim-1* vector when compared to those in the control vector-injected group (Figure 4(a)). Additionally, the severity score was 2.83 ± 0.41 before and 2.83 ± 0.75 at 1 week in the control vector group and 2.67 ± 1.21 at 2 weeks after the first injection of control vector (*n* = 6). The severity score in the *Tim-1* vector group was 3.17 ± 0.75 before injection, 1.50 ± 1.22 at 1 week after injection (*P* = 0.004), and 1.33 ± 1.51 at 2 weeks after injection in BD mice (*P* = 0.03, *n* = 6) (Figure 4(b)). The severity score decreased after injecting the *Tim-1* vector, whereas the control vector injection did not result in a difference.

Two weeks after the first administration of the *Tim-1* vector to BD mice, isolated LN and PBMCs were analyzed for Tim-1^+^ cells by FACS. In LN, the frequencies of Tim-1^+^ cells in *Tim-1* vector injected group were slightly higher compared to those in the control vector injected group (Con versus Tim-1 (%): 10.3 ± 1.7 (*n* = 4) versus 11.9 ± 3.2 (*n* = 5), *P* = 0.19). However, CD4^+^Tim-1^+^ and CD8^+^Tim-1^+^ cells were similar to those in the control vector injected group (Figure 4(c)). The frequencies of Tim-4^+^ cells were also not different (Figure 4(d)). In PBMCs, Tim-1^+^ cells were slightly lower in the *Tim-1* vector injected group, but CD4^+^Tim-1^+^, CD8^+^Tim-1^+^, and Tim-4^+^ cells were not different (Figures 4(c)-4(d)). Interestingly, CD4^+^ T cells in the Tim-1 vector injected group were slightly higher than those in the control vector injected group (Con versus Tim-1 (%): 21.3 ± 10.7 (*n* = 8) versus 27.6 ± 13.7 (*n* = 8), *P* = 0.36) (Figure 4(c)).

### 3.4. *Tim-1* Vector Administration Affects the Regulatory Cellular Phenotypes

**Figure 5:**
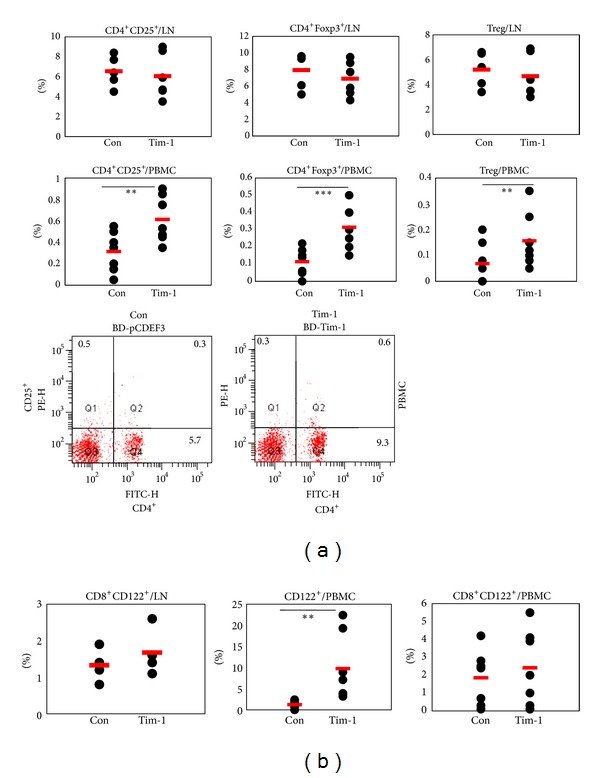
The frequencies of Treg cells in PBMCs were affected in *Tim-1* vector injected Behçet's disease (BD) mice. Two types of Treg cells were confirmed in LN and PBMCs after *Tim-1* administration to BD mice. (a) The frequencies of CD4^+^CD25^+^, CD4^+^Foxp3^+^, and Treg cells were compared between the control and *Tim-1* vector injected groups. Bottom panel shows CD4^+^CD25^+^ in PBMCs by FACS. (b) CD8^+^CD122^+^ T cells tended to be upregulated compared to those in the control group, but the difference was not significant. CD122^+^ cells increased significantly in the *Tim-1* vector injected group (*n* = 5–8) (**P < 0.05, ***P < 0.01). Con: control vector injection to BD mice, Tim-1: *Tim-1* vector injection to BD mice.

Several cellular phenotypes in the LN and PBMCs were analyzed in BD mice after administering the* Tim-1* vector. The frequencies of CD4^+^CD25^+^, CD4^+^Foxp3^+^, and CD4^+^CD25^+^Foxp3^+^ (regulatory T, Treg) cells in LN were not significantly changed in the *Tim-1* vector compared to those in the control vector injected group (Con (*n* = 5) versus Tim-1 (*n* = 6) (%): CD4^+^CD25^+^, 6.5 ± 1.6 versus 6.1 ± 2.3,  *P* = 0.35; CD4^+^Foxp3^+^, 5.3 ± 1.5 versus 4.7 ± 1.7, *P* = 0.29; Treg, 5.2 ± 1.4 versus 4.7 ± 1.7, *P* = 0.3) (Figure 5(a)). But, in PBMCs, CD4^+^CD25^+^, CD4^+^Foxp3^+^, and Treg cells in the *Tim-1* vector injected group were significantly higher than those in the control vector injected group (Con (*n* = 8) versus Tim-1 (*n* = 8) (%): CD4^+^CD25^+^, 0.31 ± 0.18 versus 0.61 ± 0.21,  *P* = 0.01; CD4^+^Foxp3^+^, 0.10 ± 0.10 versus 0.31 ± 0.12, *P* = 0.004; Treg, 0.04 ± 0.08 versus 0.16 ± 0.10, *P* = 0.03) (Figure 5(a)).

CD8^+^CD122^+^ T cells are newly identified, regarded as Treg cells [31], and are involved in anti-inflammatory responses [32]. Another type of Treg cells, CD8^+^CD122^+^ T cells, were also analyzed in LN of the *Tim-1* vector injected group (Con (*n* = 5) versus Tim-1 (*n* = 6) (%): 1.3 ± 0.3 versus 1.6 ± 0.7,  *P* = 0.25) (Figure 5(b)). In PBMCs, CD122^+^ and CD8^+^CD122^+^ T cells in the *Tim-1* vector injected group were also higher than those in the control vector injected group (Con (*n* = 8) versus Tim-1 (*n* = 8) (%): CD122^+^, 1.2 ± 1.0 versus 9.8 ± 7.9,  *P* = 0.04; CD8^+^CD122^+^, 1.9 ± 1.5 versus 2.4 ± 2.1,  *P* = 0.58).

Our study indicated that the *Tim-1* vector upregulated Treg cells and CD122^+^ cells in BD mice. Up-regulation of these cellular phenotypes may be involved in improved BD-like symptoms after injection of the Tim-1 vector.

### 3.5. Proinflammatory Cytokines are Downregulated by *Tim-1* Vector Administration in BD Mice

**Figure 6:**
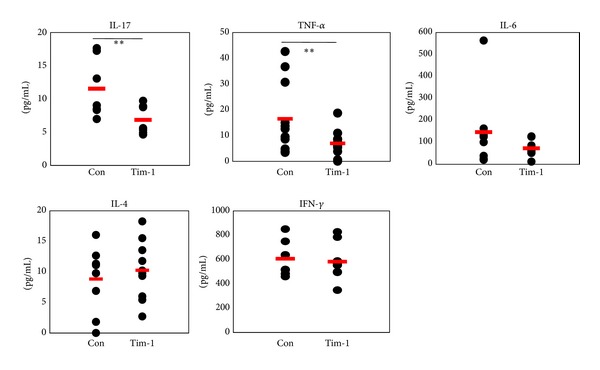
Administration of the *Tim-1* vector decreased proinflammatory cytokines in Behçet's disease (BD) mice. Serum was isolated from blood at 2 weeks after the first injection of the *Tim-1* vector. The levels of interleukin (IL)-17, tumor necrosis factor (TNF)-*α*, IL-6, interferon (IFN)-*γ*, and IL-4 were analyzed in the sera of *Tim-1* and control vector injected BD mice by ELISA (*n* = 7~11) (**P < 0.05). Con: control vector injection to BD mice, Tim-1: *Tim-1* vector injection to BD mice.

Sera were analyzed by ELISA at 2 weeks after *Tim-1* vector administration into BD mice to determine the levels of cytokines. The IL-17 level in *Tim-1* vector injected group was significantly lower than that in the control group (Con (*n* = 7) versus Tim-1 (*n* = 7) (pg/mL): 11.53 ± 4.45 versus 6.84 ± 2.17, *P* = 0.03). TNF-*α* also decreased significantly in the *Tim-1* vector injected group compared to that in the control group (Con (*n* = 11) versus Tim-1 (*n* = 11) (pg/mL): 16.5 ± 13.8 versus 6.9 ± 5.1, *P* = 0.04). The IL-6 level in the *Tim-1* vector injected group decreased (Con (*n* = 8) versus Tim-1 (*n* = 8) (pg/mL): 145.7 ± 176.9 versus 72.2 ± 38.8, *P* = 0.27). In contrast, IL-4 increased slightly in the *Tim-1* vector injected group (Con (*n* = 8) versus Tim-1 (*n* = 9) (pg/mL): 9.3 ± 5.4 versus 10.8 ± 5.0, *P* = 0.53). But, IFN-*γ* levels did not differ between the *Tim-1* and control vector injected group (Figure 6). Consequently, these results indicate that the *Tim-1* vector might downregulate proinflammatory cytokines in BD mice.

### 3.6. Tim-4 siRNA Treatment Downregulated the Expression of Tim-4 in Normal Healthy Mice

**Figure 7:**
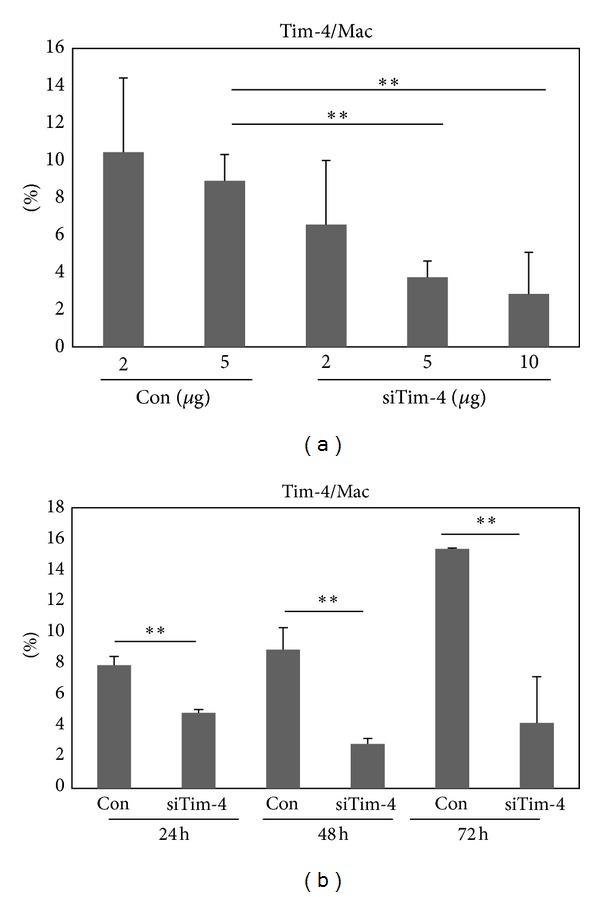
The expression of Tim-4^+^ cells was downregulated after siTim-4 treatment. Tim-4 siRNA was administered intraperitoneally and the frequencies of Tim-4^+^ cells were analyzed in peritoneal macrophages in normal (Nor) mice by FACS. (a) siTim-4 2, 5, and 10 *μ*g/mouse or negative control siRNA (Con) (2 and 5 *μ*g/mouse) were injected intraperitoneally into Nor mice. (b) Five *μ*g of siTim-4 was injected, and the frequencies of Tim-4^+^ peritoneal macrophages were analyzed 24, 48, and 72 hours later. Scrambled siRNA was used as the negative control (*n* = 3-4) (**P < 0.05). Con: control siRNA injection into normal mice, siTim-4: Tim-4 siRNA injection to normal mice, Mac: peritoneal macrophage.

In BD mice, the frequencies of Tim-4^+^ cells were higher than those in Nor and BDN mice in LN cells and peritoneal macrophages (Figure 2). siTim-4 was injected into Nor mice intraperitoneally to downregulate Tim-4^+^ cells, and the frequencies of Tim-4^+^ cells were analyzed in peritoneal macrophages by FACS. Tim-4 siRNA (siTim-4) at 2, 5, or 10 *μ*g/mouse or negative control (NC) scrambled siRNA (2 and 5 *μ*g/mouse) was injected (NC: 5 *μ*g versus siTim-4–2, 5, and 10 *μ*g (*n* = 4): 8.9 ± 1.4% versus 6.6 ± 3.4% (*P* = 0.42), 3.7 ± 0.9% (*P* = 0.013), 2.8 ± 2.2% (*P* = 0.04)) (Figure 7(a)). siTim-4 downregulated Tim-4^+^ macrophages dose dependently and showed a significant difference in the 5 *μ*g and 10 *μ*g administered groups. Therefore, 5 *μ*g injections were used for the following experiment. To determine the time-dependent efficacy of Tim-4 siRNA, 5 *μ*g of siTim-4 was injected, and the frequencies of Tim-4^+^ macrophages were analyzed by FACS after 24, 48, and 72 hours. siTim-4 significantly downregulated Tim-4^+^ macrophages until 72 hours compared to that of NC. The frequencies of Tim-4^+^ at 48 hours were lowest (NC versus siTim-4 (*n* = 2): 24 h, 7.9 ± 0.6% versus 4.9 ± 0.2%, *P* = 0.02; 48 h, 8.9 ± 1.4% versus 2.9 ± 0.4%, *P* = 0.03; 72 h, 15.4 ± 0.1% versus 4.2 ± 3.0%, *P* = 0.03) (Figure 7(b)).

### 3.7. Administration of siTim-4 Changes BD-Like Symptoms

**Figure 8:**
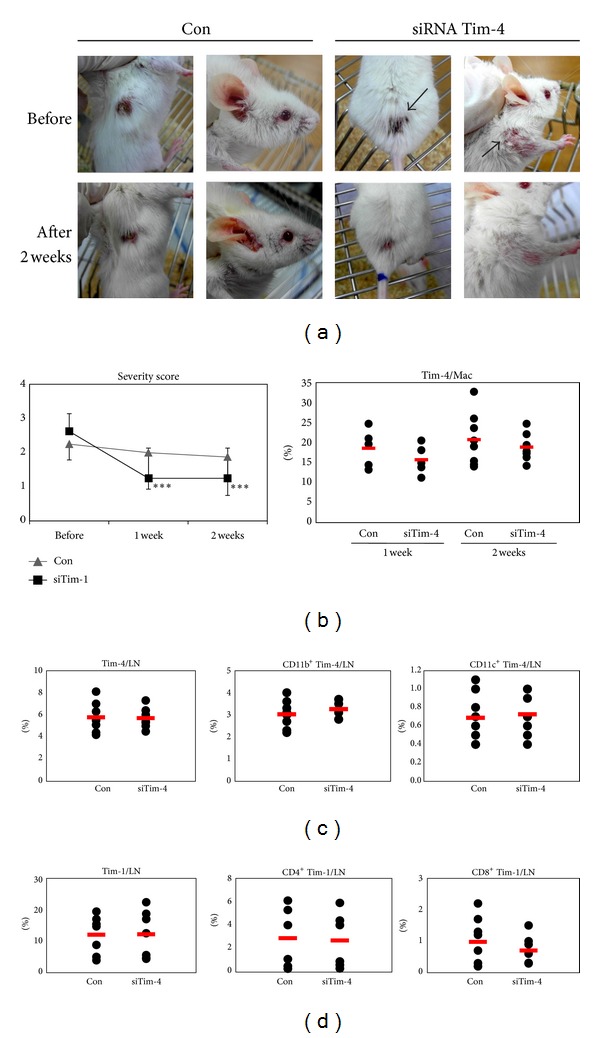
Behçet's disease (BD)-like symptoms were changed after siTim-4 administration. Five *μ*g/mouse of Tim-4 siRNA was injected intraperitoneally three times at 2 day intervals into BD mice and they were observed for 2 weeks. (a) Photographs of mice taken before and at 1 and 2 weeks after treatment with siTim-4 and the control treated group (Con: negative control siRNA treated groups). (b) The severity score was compared before and at 1 and 2 weeks after treatment between the siTim-4 and control treated groups. The frequencies of Tim-4^+^ cells in peritoneal cavity were compared between the siTim-4 and control treated groups. (c, d) The frequencies of Tim-4^+^, CD11b^+^Tim-4^+^, CD11c^+^Tim-4^+^, Tim-1^+^, CD4^+^Tim-1^+^, and CD8^+^Tim-1^+^ cells in LN were compared to the siTim-4 and control treated groups (*n* = 7-8) (**P < 0.05). Con: negative control injection to BD mice, siTim-4: Tim-4 siRNA injection to BD mice, LN: lymph node.

Five *μ*g of siTim-4 was injected intraperitoneally three times at 2 day intervals into BD mice and the mice were observed for 2 weeks (Figure 8(a)). After administration of siTim-4, BD-like symptoms, such as skin and genital ulcers, were compared to the control group. In the negative control group, the severity score was 2.25 ± 0.46 before, 2.00 ± 1.07 at one week (*P* = 0.35), and 1.88 ± 1.13 at 2 weeks after the first injection of BD mice (*P* = 0.28, *n* = 8). In the siTim-4 treated group, the score was 2.63 ± 0.52 before, 1.25 ± 0.81 at 1 week (*P* = 0.001), and 1.25 ± 0.89 at 2 weeks after injection into BD mice (*P* = 0.001, *n* = 8) (Figure 8(b)). At 1 and 2 weeks after the first administration of siTim-4 to BD mice, macrophages were isolated from the peritoneal cavity and analyzed for Tim-4 by FACS. The frequencies of Tim-4^+^ cells at 1 and 2 weeks after siTim-4 treatment tended to be lower than those in the negative control group, but the difference was not significant (1 week: 18.6 ± 4.8% versus 15.7 ± 3.7%, *P* = 0.31, 2 weeks: 20.7 ± 6.5% versus 18.8 ± 3.3%, *P* = 0.42) (Figure 8(b)). However, in LN, Tim-4^+^, CD11b^+^Tim-4^+^, CD11c^+^Tim-4^+^, Tim-1^+^, CD4^+^Tim-1^+^, and CD8^+^Tim-1^+^ cells in the siTim-4 treated group were similar to those in the control treated group at 1 week after the first siTim-4 injection (Figures 8(c)-8(d)). Even at 2 weeks after the first injection, those markers were not different (data not shown). In these results, BD-like symptoms improved with siTim-4 treatment and decreased the severity score.

### 3.8. Treg Cells are Upregulated in siTim-4 Treated BD Mice

**Figure 9:**
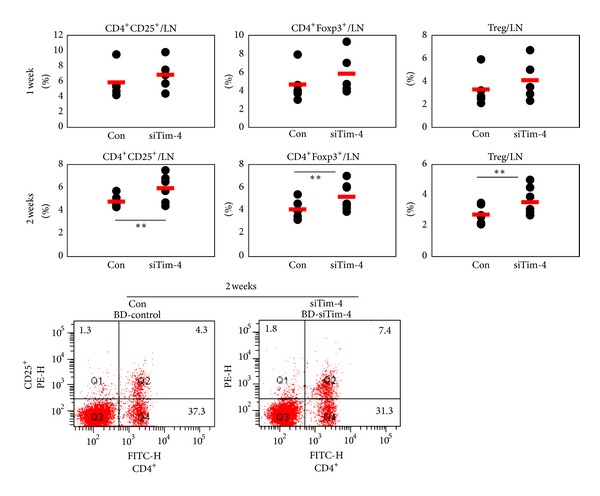
The frequencies of Treg cells were upregulated after treatment with siTim-4 in Behçet's disease (BD) mice. Treg cells were confirmed by flow cytometry in lymph nodes of BD mice after treatment with siTim-4. At 1 and 2 weeks after the first treatment with siTim-4, the frequencies of CD4^+^CD25^+^, CD4^+^Foxp3^+^, and CD4^+^CD25^+^Foxp3^+^ Treg cells were compared between the siTim-4 and control groups (negative control siRNA treated groups) (*n* = 5–7) (**P < 0.05). The bottom panel shows CD4^+^CD25^+^ in PBMCs at 2 weeks by FACS dot plot. Con: negative control siRNA injection to BD mice, siTim-4: Tim-4 siRNA injection to BD mice.

The frequencies of CD4^+^CD25^+^, CD4^+^Foxp3^+^, and Treg (CD4^+^CD25^+^ Foxp3^+^) cells were analyzed by FACS at 1 and 2 weeks after the first treatment with siTim-4 in BD mice (Figure 9). After 1 week, Treg cells increased slightly in siTim-4 treated BD mice compared with those in the NC treated group (NC versus siTim-4: CD4^+^CD25^+^, 5.82 ± 2.01% versus 6.84 ± 2.03%, *P* = 0.45, *n* = 5; CD4^+^Foxp3^+^, 4.64 ± 1.9% versus 5.82 ± 2.4%, *P* = 0.41, *n* = 5; Treg, 3.28 ± 1.57% versus 4.08 ± 1.73%, *P* = 0.47, *n* = 5). After 2 weeks, Treg cells were significantly higher in siTim-4 treated BD mice than those in the NC treated group (NC versus siTim-4: CD4^+^CD25^+^, 4.8 ± 0.6% versus 5.9 ± 1.2%, *P* = 0.03, *n* = 6; CD4^+^Foxp3^+^, 4.1 ± 0.7% versus 5.2 ± 1.2%, *P* = 0.03, *n* = 7; Treg, 2.7 ± 0.5% versus 3.5 ± 0.9%, *P* = 0.04, *n* = 7). These results suggest that the increased number of Treg cells is associated with knock down of Tim-4 in BD mice.

### 3.9. Treatment with siTim-4 Decreases Serum Levels of IL-17 in BD Mice

**Figure 10:**
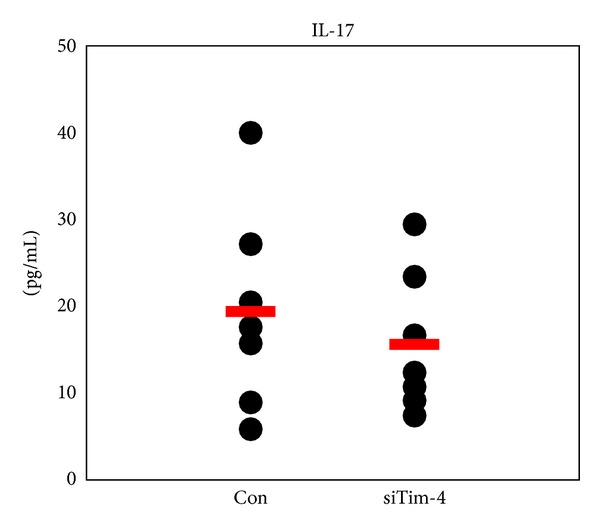
Administration of siTim-4 decreased serum level of IL-17 in Behçet's disease (BD) mice. Serum obtained at 2 weeks after the first injection of siTim-4 and control (Con) in BD mice. The level of IL-17 was analyzed by ELISA (*n* = 8). Con: negative control siRNA injection to BD mice, siTim-4: Tim-4 siRNA injection to BD mice.

After administering siTim-4 to BD mice, the serum level of IL-17 was analyzed by ELISA and compared with NC siRNA treated BD mice. The level of IL-17 tended to decrease in the siTim-4 treated group compared to that in the NC treated group, but the difference was not significant (NC versus siTim-4 (*n* = 8): 19.4 ± 11.5 pg/mL versus 15.6 ± 8.1 pg/mL, *P* = 0.25) (Figure 10).

## 4. Discussion

BD mice downregulated Tim-1 levels and upregulated Tim-4 levels in LN, SP, PBMCs, and peritoneal macrophages compared to those in BDN mice. Administration of the *Tim-1* vector upregulated the frequencies of Tim-1^+^ cells, and Tim-4 siRNA downregulated the frequencies of Tim-4^+^ cells. Administering the *Tim-1 *vector improved BD-like symptoms, such as genital and skin ulcers, and decreased the severity score. TIM-1 expression is lower in patients with active SLE compared to that in inactive patients [18]. Human TIM-1 is associated with immune dysfunction, such as atopic dermatitis, allergy, rheumatoid arthritis, asthma, and SLE [14–18]. Our data indicate that the Tim-1 related T cells phenotype did not change much after administering the* Tim-1* vector. Interestingly, the frequencies of CD4^+^ T cells in PBMCs increased in the *Tim-1* vector injected group compared to those in the control vector injected group. Actually, TIM-1 is expressed on CD4^+^ T cells after activation and its expression is preferentially sustained on Th2 but not Th1 cells [1, 7]. Xiao et al. reported that Tim-1-Fc triggers a significant increase in the frequencies of CD4^+^ T cells [33]. In our study, Treg cells were upregulated in the *Tim-1* vector injected BD group. Other Treg cells, CD8^+^CD122^+^ T cells, were also higher in PBMCs and LN of the *Tim-1 *vector injected group compared to those in the control group. CD8^+^CD122^+^ T cells are newly identified and regarded as Treg cells [31] and have an effect on anti-inflammatory responses [32]. Our results suggest that the function of Tim-1 influenced the BD-like symptoms and is associated with Treg and CD8^+^CD122^+^ T cells.

Recent studies suggest that IL-17 may play a dominant role in provoking chronic autoimmune inflammation and is considered essential for T cell-mediated colitis and promotion of inflammation [34–36]. The association of IL-17 and IL-22 was also reported in patients with BD [37, 38]. TNF-*α* overexpression has been implicated in acute and chronic inflammatory diseases, such as septic shock, bowel disease, Crohn's disease, rheumatoid arthritis, atopic dermatitis, psoriasis, and BD [39]. Overproduction of IL-6 plays a role in rheumatoid arthritis, juvenile idiopathic arthritis [40], inflammatory bowel disease [41], and SLE [42]. The anti-Tim-1 antibody (high avidity/agonistic) is a down-regulator of pro-inflammatory Th-17 cells. TIM-1-TIM-4 interaction is involved in the regulation of Th cell responses and modulation of the Th1/Th2 cytokine balance [7]. Our data also indicate that administering the *Tim-1* vector decreased proinflammatory cytokines, such as IL-17, TNF-*α*, and IL-6, but the Th2 type cytokine IL-4 was upregulated in the *Tim-1* vector injected group. Recently, upregulated cytokine IL-22 in ocular BD patients was also downregulated by anti-IL-6 and anti-TNF-*α* [37]. These results were similar to our results.

BD mice displayed markedly increased Tim-4 levels in LN and peritoneal macrophages compared to those in Nor and BDN mice. The frequencies of Tim-4^+^ cells were downregulated when Tim-4 siRNA was administered to Nor mice compared to those in the NC group. siTim-4 administration to BD mice tended to decrease Tim-4^+^ cells in peritoneal macrophages. siTim-4 administered BD mice displayed improved symptoms such as skin and genital ulcers and arthritis and showed a decreased severity score. But, Tim-4^+^ cells related T cell phenotypes were not changed after siTim-4 treatment. TIM-4 is a ligand for TIM-1 [7] and is not expressed in T cells but is exclusively expressed in antigen-presenting cells, including DCs and macrophages [9, 10]. Two groups have reported that Tim-4 deficient mice with a 129 or b6 background develop little or no autoimmunity [9, 43]. In conjunction with improved symptoms, Treg cells in the siTim-4 treated group were significantly upregulated compared to those in the control group, and IL-17 level decreased. In our previous study, adoptive transfer of Treg cells to BD mice also improved BD-like symptoms [44]. In addition, the frequencies of Treg cells are lower in patients with autoimmune and inflammatory diseases, such as Crohn's disease [45], multiple sclerosis [46], and SLE [47], than those in healthy controls and inactive patients. The TIM-1-TIM-4 interaction is important for regulating T cell proliferation and modulating the Th1/Th2 cytokine balance [7]. Our data suggest that upregulated Treg cells induced by siTim-4 might contribute to improve BD-like symptoms.

In conclusion, the frequencies of Tim-1^+^ cells in BD mice were downregulated compared to those in BDN mice. Administering the *Tim-1* vector to BD mice improved the BD-like symptoms and decreased the severity score by up-regulating Treg cells and down-regulating pro-inflammatory cytokines. In addition, treatment with siTim-4 upregulated Treg cells and improved BD-like symptoms, which were related to the Tim-1 and Tim-4 expression and the Tim-1-Tim-4 interaction. Furthermore, Tim-4 siRNA downregulated the level of IL-17, which may have been involved in improving the BD-like symptoms. Consequently, regulation of Tim-1 and Tim-4 was effective for improving BD-like symptoms in mice.
